# Predicting Mild Cognitive Impairment in Type 2 Diabetes: A Machine Learning Approach

**DOI:** 10.1155/jdr/7304414

**Published:** 2025-10-19

**Authors:** Fangyi Li, Shengyi Zhao, Tianyu Wu, Sijue Yang, Yanjie Duan, Jing Sun, Wenhui Zhu, Beibei Zhai, Congcong Yu, Shihua Chen, Zhou Zhang, Wei Tang, Yan Bi

**Affiliations:** ^1^Department of Endocrinology, Endocrine and Metabolic Disease Medical Center, Nanjing Drum Tower Hospital Clinical College of Nanjing University of Chinese Medicine, Nanjing, China; ^2^Department of Endocrinology, Endocrine and Metabolic Disease Medical Center, Nanjing Drum Tower Hospital, Affiliated Hospital of Medical School, Nanjing University, Nanjing, China; ^3^Branch of National Clinical Research Centre for Metabolic Diseases, Nanjing, China; ^4^Department of Endocrinology, Yancheng Traditional Chinese Medicine Hospital, Yancheng, China; ^5^Department of Endocrinology, Geriatric Hospital of Nanjing Medical University, Nanjing, China

**Keywords:** machine learning, mild cognitive impairment, prediction model, Type 2 diabetes mellitus

## Abstract

**Background:**

Diabetes significantly increases the risk of cognitive impairment, particularly mild cognitive impairment (MCI). Early identification of individuals at risk for MCI is crucial for timely intervention. This study was aimed at developing and validating a machine learning–based model to predict MCI in patients with Type 2 diabetes (T2DM).

**Methods:**

Participants with T2DM and completed cognitive assessments were included. Feature selection was done using statistical methods and genetic programming to reduce collinearity. Six classification models were trained and evaluated using cross-validation and hyperparameter tuning. External validation was performed with cohorts from the Jiangsu DiabEtes COgnitive Dysfunction Early Diagnosis and Intervention (DECODE) study and the Third National Health and Nutrition Examination Survey (NHANES III). SHAP analysis identified key predictors, and a web interface was developed for practical application.

**Results:**

A total of 2074 participants were included. Significant predictors were education, age, GCA index (glycolipid metabolism), systolic blood pressure, eGFR, BMI, and diabetes duration. The support vector classifier (SVC) model achieved the highest performance, with an AUC of 0.74 ± 0.04, an *F*1 score of 0.62 ± 0.06, and a recall of 0.74 ± 0.09 in internal validation. External validation with the DECODE cohort yielded an AUC of 0.80, an *F*1 score of 0.80, and a recall of 0.89. NHANES III validation confirmed the model's reliability in predicting MCI risk.

**Conclusions:**

This study compared machine learning models for diagnosing MCI in T2DM patients. The SVC model demonstrated strong efficacy and accuracy, highlighting the potential of machine learning in diagnosing MCI in this population.

## 1. Introduction

The global prevalence of diabetes and cognitive dysfunction is rising, posing significant health and economic challenges. The number of individuals with diabetes is projected to reach 783 million by 2045 [1], while over 55 million people currently live with dementia worldwide, a figure expected to triple by 2050 [2]. Notably, diabetes significantly increases the risk of cognitive dysfunction, which is classified as mild cognitive impairment (MCI) or dementia based on the disease severity [3]. Data from the UK Clinical Practice Research Datalink reveal that dementia-related deaths among individuals with diabetes rose from 2% in 2001 to 16% in 2018, making it the second leading cause of death after malignancies [4]. A longitudinal study further showed that MCI in diabetic patients accelerates the onset of dementia by an average of 3.18 years [5]. The economic burden is substantial, with the global cost of dementia exceeding $1 trillion annually, and this number continues to rise [6]. Despite these alarming statistics, no approved medications can significantly slow the progression of dementia. However, early detection and interventions could prevent or delay up to 40% of dementia cases [7]. Therefore, early screening for cognitive impairment in high-risk populations, such as those with diabetes, is crucial to mitigate the growing clinical and economic impact.

Guidelines from the American Diabetes Association (ADA) and the European Society of Endocrinology (ESE) recommend routine cognitive impairment screening for diabetic patients over 65 [8]. Currently, MCI diagnosis relies heavily on neuropsychological tests, with the Montreal Cognitive Assessment (MoCA) being a commonly recommended tool [9]. However, factors like education, age, language, and cultural differences can influence test results, and clinical evaluations must be conducted by experienced clinicians, potentially increasing the healthcare workload. Additionally, neuroimaging techniques, including magnetic resonance imaging and positron emission tomography, are utilized for diagnosing MCI [10], but their limited sensitivity in detecting early cognitive decline, along with high costs, restricts their use in routine screening. While cerebrospinal fluid and serum markers show promise in diagnosing cognitive impairment [11], the biomarkers currently available in clinical practice are limited. Consequently, there is an urgent need to develop an early risk prediction model for MCI in individuals with diabetes to enable timely interventions [12].

The pathophysiology of cognitive decline in T2DM is complex, involving metabolic dysregulation, vascular dysfunction, and neurodegeneration [13]. Although several prediction models have been developed for cognitive impairment in patients with T2DM, conventional models with a limited number of variables may not optimally predict MCI [14–16]. Furthermore, these studies were often limited by small sample sizes or lack of external validation, limiting their generalizability. Machine learning (ML) algorithms, which can process high-dimensional and nonlinear data, offer the potential to improve predictive accuracy [17]. In this study, we leverage a large, comprehensive dataset that includes demographic characteristics, clinical parameters, metabolic markers, and cognitive assessments of diabetic patients, thereby enhancing the clinical relevance of our ML-driven MCI prediction models.

This study was aimed at developing an ML-based MCI prediction model for patients with T2DM, utilizing only parameters readily available during physical examination to enable early risk screening and intervention strategies for cognitive impairment.

## 2. Materials and Methods

### 2.1. Study Population

This cross-sectional study prospectively enrolled adult inpatients diagnosed with T2DM from the Department of Endocrinology at Nanjing Drum Tower Hospital over the period from January 2016 to December 2023. The study builds upon and expands a previously established cohort from earlier research [18]. Participants with T2DM (based on the ADA 2019 criteria for the diagnosis [19]), aged ≥ 40 years, and having undergone cognitive assessment were included. Exclusion criteria included (1) clinically suspected or diagnosed dementia; (2) less than 6 years of education; (3) hearing and/or visual impairment; (4) severe psychiatric disorders, particularly anxiety or depression; (5) acute diabetic complications, such as diabetic ketoacidosis, hyperosmolar hyperglycemia, or hypoglycemia-induced coma; (6) recent cardiovascular or cerebrovascular incidents occurring within the preceding 3 months; (7) systemic metabolic diseases, including Cushing's syndrome and thyroid, parathyroid, or gonadal disorders; and (8) severe chronic diseases, such as rheumatism, hemophilia, or malignancies. The study was approved by the Ethics Committee of Nanjing Drum Tower Hospital (2017-017-01, 2017-017-02, 2017-017-03, and 2017-017-04). Our study conformed to the guidelines of the 1964 Helsinki Declaration and its later amendments or comparable ethical standards. All participants provided written informed consent.

### 2.2. Clinical Trait Acquisition and Cognitive Function Evaluation

Clinical features, including demographic, laboratory, physical, and medical history data, were collected from electronic health records. Detailed descriptions and the full list of variables are provided in the Supporting Information.

General cognitive function was assessed using the Beijing version of the MoCA, for which all assessing clinicians involved in this study hold official certification. A score below 26 was used to indicate cognitive impairment, with adjustments made for educational level. Activities of daily living (ADLs) were evaluated using the Barthel ADL scale, and dementia severity was determined via the Clinical Dementia Rating Scale (CDRS). The diagnosis of MCI followed the 2018 guidelines from the American Academy of Neurology, incorporating subjective complaints, objective evidence, functional independence, and the absence of dementia. Neuropsychological assessments were conducted by trained clinicians.

The Beijing version of MoCA was applied to assess the general cognitive function. A MoCA score of < 26 points indicated objective cognitive impairment, with an additional point added to the total score for patients with less than or equal to 12 years of formal education. The participant's capacity to execute daily activities was evaluated using the Barthel ADL scale, where a score above 60 was considered indicative of basic self-care capabilities. Dementia was indicated by a CDRS score > 0.5. The aforementioned neuropsychological tests were conducted by a team of experienced clinicians. The diagnosis of MCI was based on the guidelines by the American Academy of Neurology in 2018 [20]: (1) subjective perception of cognitive decline by the patient or informant, (2) objective evidence of cognitive impairment, (3) minor impairment of complex instrumental daily living skills but maintaining independent daily living functions, and (4) absence of dementia. Subjects were then grouped according to their respective diagnoses of MCI according to the above criteria.

### 2.3. Data Cleaning: Scaling and Missing Data Imputation

The k-nearest neighbor (KNN) method was applied to impute missing data, with values for incomplete features estimated using the closest observations in the dataset. Subsequently, the data were randomly split into training and validation subsets at a 7:3 ratio. Both sets were normalized using the StandardScaler from the scikit-learn library.

### 2.4. Feature Engineering: Genetic Programming and Feature Selection

Due to the high dimensionality of the data, genetic programming was employed in the ML pipeline to construct a combined feature set from laboratory data [21]. A population of 1000 terminals was initialized using the half-and-half method, and the top 50 programs with the best correlation to the original datasets were selected for crossover and reproduction. This process was iterated for 1000 generations, and the most suitable program was selected as the final representation of the laboratory data.

To select the most relevant features for model development, a model-based feature selection process was performed. The dataset was trained using a random forest (RF) classifier with default parameters, and the top 10 most important features were used in the training and validation processes.

### 2.5. Model Development and Fine-Tuning

Six ML models were initially trained, including logistic regression (LR), RF, adaptive boosting (AdaBoost), support vector classifier (SVC), categorical boosting (CatBoost), and multilayer perceptron (MLP), all with default parameters. The top three models, based on cross-validation performance, were further optimized using Bayesian hyperparameter tuning implemented with Optuna [22]. The best models were selected based on their area under the receiver operating characteristic (ROC) curve.

### 2.6. Model Evaluation and Validation

Models were evaluated using a set of four parameters, including accuracy, precision, recall (sensitivity), and *F*1 score (the harmonic mean of precision and recall), which were assessed on the test set. The whole dataset was sampled by stratified K-fold to perform cross-validation. Confidence intervals were computed with the mean and standard deviation of the metrics during cross-validation. Moreover, we examined the importance of each model's features using SHapley Additive exPlanations (SHAP) [23].

To further validate the model, we tested it on two independent datasets: the DECODE cohort (Cohort 1) and the NHANES III cohort (Cohort 2). Model performance on the validation sets was assessed using the same process, and predicted probabilities were compared with cognitive test results to evaluate the model's alignment with cognitive assessments.

### 2.7. Statistics

Continuous data were presented as mean ± standard deviation or median (interquartile range) and compared using the *t*-test. Categorical data were expressed as counts and percentages and analyzed using the chi-squared test. Analyses were performed in SPSS v26.0, with *p* < 0.05 indicating significance.

The model's performance was evaluated on internal and external datasets using metrics for discrimination and calibration. Discrimination was measured via the area under the curve (AUC), while calibration was assessed with plots and Brier scores. Classification metrics, including accuracy, precision, recall, and the *F*1 score (useful for imbalanced datasets), were derived from a confusion matrix. Decision curve analysis (DCA) quantified clinical utility across threshold probabilities, and SHAP plots provided insights into the influence of predictors on model outputs.

All ML algorithms were implemented using the scikit-learn library (v1.3.0) in Python (v3.10.14). Model visualizations were generated using the SHAP library (v0.44.0). A web-based application was developed using Dash (v2.16.1) and Plotly (v5.19.0).

## 3. Results

### 3.1. Baseline Characteristics

**Figure 1:**
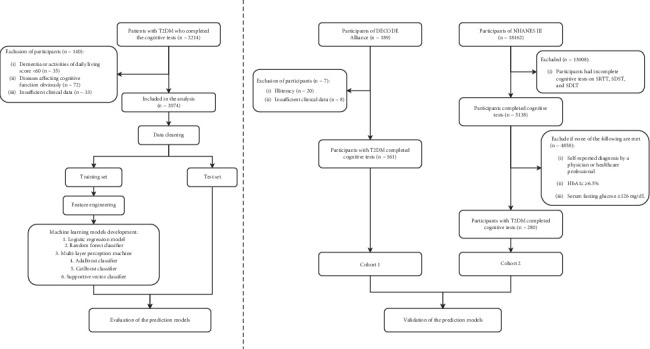
Schematic framework for developing a machine learning–based model for MCI diagnosis.

The study design and data analysis process are shown in Figure 1. Among the 2074 individuals with T2DM, 724 were classified into the MCI group, while 1350 were in the normal cognitive state (NCS) group.  Table 1 summarizes the clinical and demographic features of both groups. The MCI group had a higher average age, lower education level, more females, longer diabetes duration, and higher systolic blood pressure (SBP) compared to the NCS group (*p* < 0.05). Biochemically, the MCI group exhibited significantly higher fasting plasma glucose (FPG) (8.3 ± 2.7 vs. 8.5 ± 2.7; *p* = 0.026), HDL-C (1.2 ± 0.3 vs. 1.3 ± 0.4; *p* < 0.001), and ApoA (1.1 ± 0.2 vs. 1.2 ± 0.2; *p* < 0.001), as well as lower levels of postprandial C-peptide (PCP) (1804.0 ± 1026.1 vs. 1654.4 ± 971.5; *p* = 0.001), TG (1.7 ± 1.3 vs. 1.6 ± 1.0; *p* = 0.018), and eGFR (119.7 ± 31.8 vs. 116.6 ± 32.0; *p* = 0.019). Additionally, the MCI group had a higher prevalence of diabetic peripheral neuropathy, peripheral vascular disease, cardiovascular disease, cerebrovascular disease, hypertension, and osteoporosis (*p* < 0.05). There were no significant differences in family history between the two groups. Participants were randomly assigned to training (*n* = 1451) and validation (*n* = 623) datasets for model development and testing, with no significant differences between the sets (*p* > 0.05), confirming their comparability (Table S1).

### 3.2. Statistics-Based and Model-Based Feature Selection

To reduce the dataset's dimensionality, we applied both statistics-based and model-based feature selection strategies. First, Pearson's correlation coefficient (PCC) was calculated and variables with a PCC > 0.5 were removed (Figure S1A). Next, a genetic programming model was applied to integrate laboratory data in the training sets. Using mean absolute error (MAE), the optimal representation of the lab data was derived after 100 generations of self-evolution, with a fitness metric based on Spearman's correlation coefficient. The final formula of the representation is (PCP + TC) + 2∗(TG∗TC) + log(FPG) − ApoA. Further variable selection was conducted using a model-based strategy, where a RF classifier was trained with default parameters. The top 10 most important variables were selected (Figure S1B). Ultimately, variables including age, education, eGFR, SBP, BMI, T2DM duration, and the previously derived representation index were incorporated into model development.

### 3.3. Classification Model Selection and Evaluation

**Figure 2:**
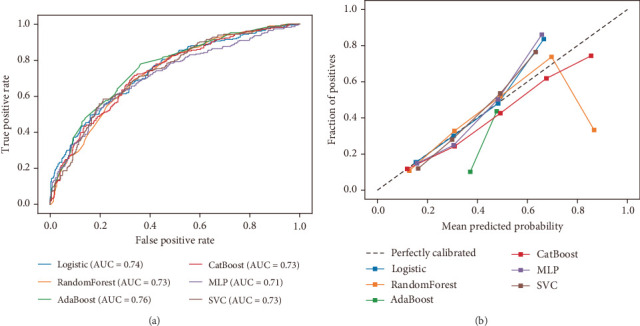
Model performance of baseline models on internal test dataset. (a) ROC curves of baseline models. (b) Calibration curves of baseline models. Abbreviations: ROC, area under the curve; AdaBoost, adaptive boosting; CatBoost, categorical boosting; MLP, multilayer perceptron; SVC, supportive vector classifier.

Initially, we trained six classification models with default parameters and evaluated their performance using fivefold cross-validation, summarized in Figure 2 and Table 2. The LR model served as the baseline.  Figure 2a displays the mean cross-validated ROC AUC for each model. AdaBoost achieved the highest AUC (AUC = 0.75 ± 0.02), followed by CatBoost (AUC = 0.74 ± 0.02), SVC (AUC = 0.73 ± 0.03), LR (AUC = 0.73 ± 0.01), RF (AUC = 0.73 ± 0.03), and MLP (AUC = 0.70 ± 0.03). Calibration curves for the LR, CatBoost, MLP, and SVC models demonstrated a closer alignment with the ideal calibration line, indicating better model fitness (Figure 2b). The performance of these models in predicting MCI in patients with T2DM was further evaluated across multiple metrics, including *F*1 score, recall, accuracy, and precision (Table 2). Among the models tested, the SVC model delivered the highest *F*1 score (0.62 ± 0.06) and recall (0.74 ± 0.09), outperforming the CatBoost model, which had an *F*1 score of 0.61 ± 0.02 and recall of 0.64 ± 0.02.

**Figure 3:**
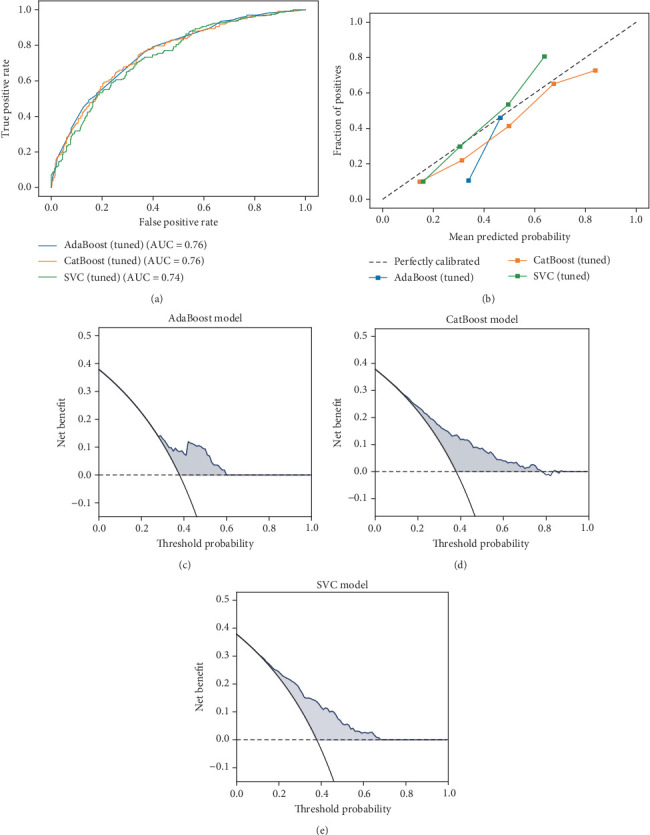
Model performance of tuned models on internal test dataset. (a) ROC curves of tuned models. (b) Calibration curves of tuned models. (c–e) Decision curve analysis of tuned models. Abbreviations: ROC, area under the curve; AdaBoost, adaptive boosting; CatBoost, categorical boosting; SVC, supportive vector classifier.

After performing ROC analysis and assessing the optimal balance between recall and *F*1 score, we identified AdaBoost, CatBoost, and SVC as the top-performing models. These models were then fine-tuned using Optuna for hyperparameter optimization. As shown by the ROC curves in Figure 3a, the tuned models demonstrated similar performance, with AUC values between 0.74 and 0.76. Calibration plots (Figure 3b) indicated that the prediction probabilities of the CatBoost and SVC models were highly concordant with observed MCI outcomes, further supported by lower Brier scores. To assess the clinical utility of the models, DCA was performed, which revealed that the CatBoost and SVC models provided superior net benefits compared to the others across a range of threshold probabilities (Figures 3c, 3d, and 3e). Notably, the SVC model achieved the highest *F*1 score (0.63 ± 0.04) and recall (0.75 ± 0.06) after tuning (Table 3), highlighting its effectiveness in identifying MCI cases among patients with T2DM. Based on these results, the SVC model emerged as a suitable algorithm for this task.

### 3.4. External Validation of the ML Model With the NHANES III and DECODE Cohort

**Figure 4:**
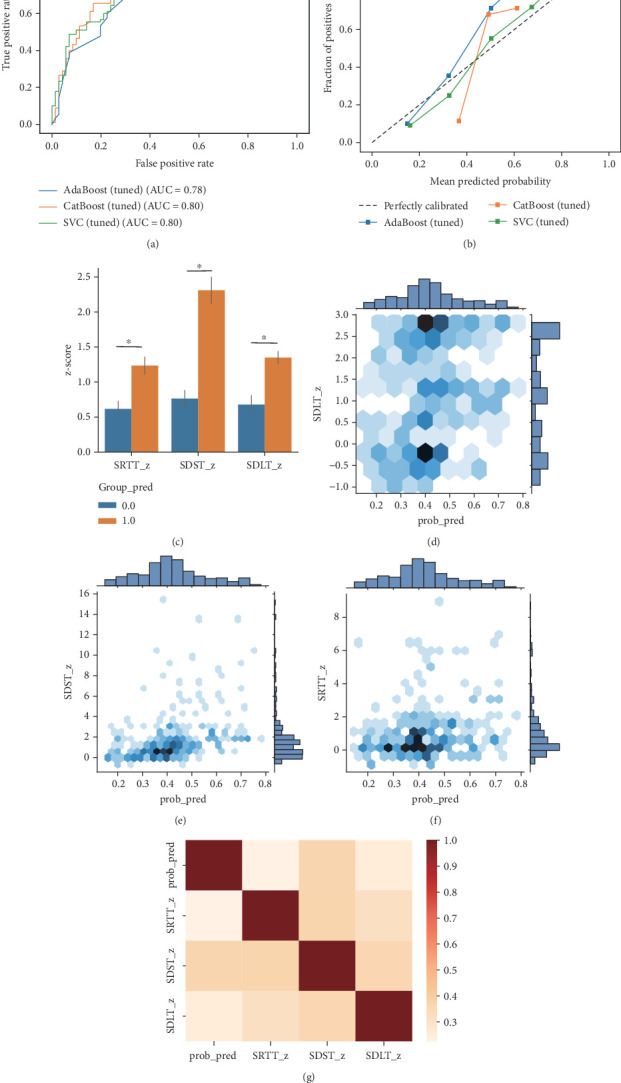
Model performance of tuned models on external validation datasets. (a) ROC curves of tuned models on Validation Cohort I; (b) curves of tuned models on Validation Cohort I; (c) comparison of cognitive test scores between the predicted MCI and control groups on Validation Cohort II; (d–f) contour plot to evaluate the correlation between cognitive test scores and predicted probabilities of MCI on Validation Cohort II; (g) correlation heatmap between cognitive test scores and predicted probabilities of MCI on Validation Cohort II.

To evaluate the performance of our prediction models in external validation datasets, we applied them to diabetic populations in Cohort 1 (DECODE) and Cohort 2 (NHANES III), with baseline characteristics summarized in Table S2. In the external validation of Cohort 1, the SVC model achieved the highest AUC of 0.80, demonstrating good calibration (Figure 4a,b). Moreover, the SVC model exhibited superior performance, with an *F*1 score and recall of 0.80 and 0.89, respectively, reinforcing its robustness in predicting MCI in patients with T2DM. In Cohort 2, the SVC model's predictions indicated a significant cognitive performance gap between the high-risk and low-risk groups (*p* < 0.05) (Figure 4c). Figures 4d, 4e, and 4f show that participants with higher scores on cognitive assessments correspondingly had higher model scores, suggesting a greater predicted risk of MCI. This positive correlation supports the predictive validity of the model, highlighting its ability to identify individuals with early cognitive decline based on their cognitive test performance. Additionally, a heatmap representing the correlation between the model-predicted scores and cognitive test outcomes is shown in Figure 4g, further aligning the model with cognitive assessments.

### 3.5. SHAP of the Classification Model

**Figure 5:**
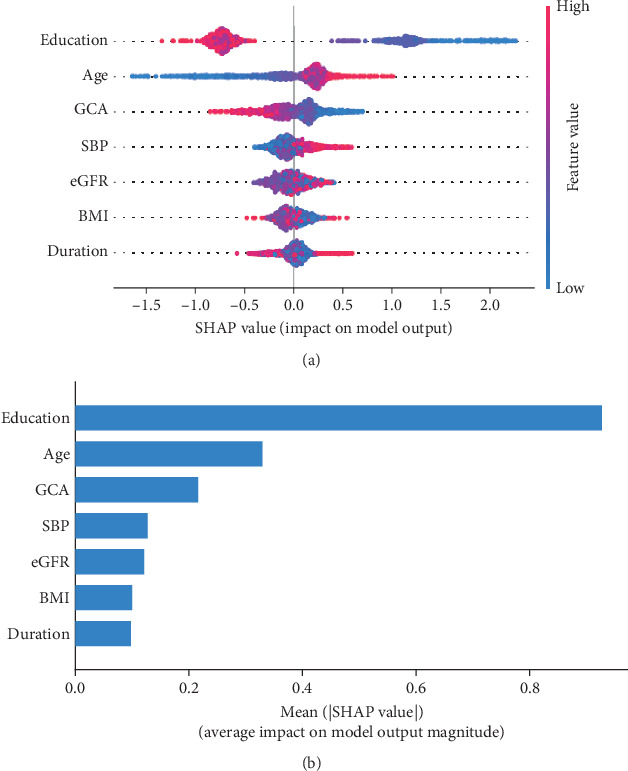
SHAP analysis of the model: (a) beeswarm plot of prediction variables; (b) relative feature importance of prediction variables. Note: *GCA index*: a composite measure of glucose–insulin (FPG, C-peptide) and lipid (TG, TC, ApoA) dysregulation associated with cognitive risk.

Using SHAP analysis, we explained individual predictions and measured the global importance of features.  Figure 5a presents a SHAP summary plot, illustrating the impact of each predictor, with value magnitude indicated by color gradients and the direction of influence on the horizontal axis (indicating the likelihood of T2DM-related MCI). Each point represents a patient's eigenvalue and corresponding SHAP value. For instance, participants with low education (represented in red) are more likely to experience cognitive dysfunction (left), while those with higher education (blue) tend to be cognitively healthy (right).  Figure 5b displays a bar plot of feature importance assessed by SHAP values, with education and age identified as the most significant variables. Our integrated index, which reflects glucose and lipid metabolism, ranked third in predicting MCI, followed by SBP, eGFR, BMI, and diabetes duration.

### 3.6. Model Presentation

The final model, developed using the training cohort, was deployed via a user-friendly web interface to facilitate broader application. A screenshot of the generalized model is provided in Figure S2. The web application is available online at https://diabcogrisk.cpolar.cn.

## 4. Discussion

We developed an ML-based diagnostic model to predict MCI in patients with T2DM, utilizing a large and comprehensive dataset. Our comparison of multiple ML models demonstrated the efficacy and accuracy of the proposed diagnostic algorithm. The model's robustness and applicability were further validated through external testing across diverse datasets. Additionally, SHAP analysis identified seven key variables influencing MCI risk, offering new insights into the underlying factors driving cognitive decline in this population. To support clinical application, we also developed an online calculator, *DiabCogRisk*, which estimates individual risks of cognitive impairment in patients with T2DM, facilitating early detection and timely intervention to improve patient outcomes.

While numerous studies have established risk prediction models for cognitive impairment in the elderly population [24, 25], models on MCI in patients with diabetes are scarce. Three cross-sectional studies [24, 26, 27] proposed nomograms for MCI risk prediction, incorporating factors like education, age, HbA1c, duration of diabetes, physical activity, and depression. These models are limited by small sample sizes and lack of external validation, reducing their broader applicability. Furthermore, these models rely on a narrow set of predictors, which may not fully capture the complex, multifactorial nature of MCI development in diabetes. In contrast, our study significantly expands upon previous research by including the largest single-center investigation of cognitive dysfunction in T2DM to date [18]. We also integrated a comprehensive range of variables, encompassing demographic variables, medical history, physical examination results, and laboratory tests—factors readily available during routine clinical evaluations. Moreover, the thorough internal and external validation conducted in our study strengthens the reliability and generalizability of the model.

Recently, ML algorithms have demonstrated efficacy in handling numerous variables, with increasing applications in detecting and predicting cognitive dysfunctions [28] in capturing complex, nonlinear relationships between predictors and outcomes, which are often overlooked by traditional statistical methods. In this retrospective study, we applied six ML algorithms to develop predictive models for MCI in patients with T2DM. During the initial stages of model development, the model's performance was limited by the dataset's multidimensionality [29]. A common approach to this issue is the application of dimensionality reduction algorithms, such as principal component analysis and linear discriminative analysis, which transform data into a latent space for further classification tasks [30]. However, these algorithms significantly reduced the information within the dataset. To mitigate information loss, we employed a genetic programming–based transformer to calculate a combinational score that encapsulates the lab data [31]. The algorithm generated an index integrating the glucose-insulin profile (including FPG and C-peptide) with the lipid profile (comprising TG, TC, and ApoA), which we termed the GCA index. Previous research has demonstrated that glucose–lipid metabolic dysfunction is closely linked to cognitive decline in individuals with T2DM. For instance, data from the UK Biobank revealed a U-shaped association between total cholesterol levels and dementia risk [32]. Moreover, cross-sectional studies have identified elevated ApoA and FPG levels as significant risk factors for cognitive impairment in patients with T2DM [33, 34]. In accordance with the most recent findings [35], we also identified a positive correlation between higher HDL-C level and risk of developing cognitive impairment in T2DM. Though contradictory findings present [36], the association between glucolipid metabolism and cognitive decline persisted, leading for our development of an index reflective of the glucolipid metabolic state. Our study is the first to design the GCA index to provide a more comprehensive assessment of the relationship between metabolic dysfunction and cognitive impairment. By incorporating these variables, we aimed to improve the model's capacity to capture the metabolic disturbances associated with cognitive decline in patients with T2DM. This novel use of the GCA index underscores the potential of ML algorithms to enhance the early detection of cognitive dysfunction in T2DM, enabling more targeted intervention strategies.

Explainable artificial intelligence (XAI) methods, such as LIME and SHAP, enhance the interpretability and transparency of predictive models for cognitive dysfunction by providing local linear approximations [37]. In this study, SHAP analysis identified education, age, the GCA index, SBP, eGFR, BMI, and diabetes duration as significant predictors of cognitive impairment in individuals with T2DM. Consistent with previous findings, education and age were the strongest predictors of MCI [38, 39]. Additionally, SBP also emerged as a key predictor, aligning with research indicating that intensive SBP control may improve cognitive outcomes, thus facilitating more targeted interventions for MCI [40]. A significant nonlinear relationship between eGFR and cognitive dysfunction has been reported, suggesting that the link between kidney disease and cognitive impairment may be underestimated. Our study corroborates this finding, highlighting the importance of including eGFR as a variable in predictive models [41]. Weight changes in individuals with diabetes, whether weight loss or gain, are associated with an increased risk of cognitive impairment [42], a result consistent with our findings. Low BMI [43] may be related to malnutrition, metabolic disorders, and mental health issues, while weight gain [7] can exacerbate insulin resistance and other metabolic disturbances, further impairing cognitive function. Diabetes duration has also been widely recognized as a risk factor for cognitive decline. Overall, high-risk individuals may benefit from aggressive management of modifiable risk factors, including tight control of blood glucose, blood pressure, and lipid levels, as well as maintaining optimal eGFR and BMI. While each predictor has been individually validated in prior studies, this study is the first to integrate 11 distinct variables into a unified model for MCI risk prediction in T2DM, significantly improving the model's accuracy and reliability.

Furthermore, external validation is essential to ensure the generalizability and robustness of predictive models. In this study, we validated the performance of our SVC model using the DECODE cohort, which includes a geographically diverse population, thereby increasing the model's representativeness. This validation yielded an AUC of 0.80, with a high *F*1 score and recall, indicating reliable predictive performance. Further validation with the NHANES III cohort, which includes participants from various racial and ethnic backgrounds, demonstrated the model's robustness across diverse populations. Significant differences in cognitive performance between high- and low-risk individuals, along with a positive correlation between model-predicted scores and cognitive test outcomes, provide strong evidence of the model's ability to detect early cognitive decline. This model demonstrates significant potential for population-based screening to identify individuals at elevated risk of dementia onset, thereby enabling timely preventive interventions. Moreover, to facilitate clinical application, we developed a portable application to enhance the acceptability of our model. Most previous research focused on the development of the model, while the assessment tools were either time-consuming or hard to use [18, 24]. To address this, we developed an application with the ability to merge with the current electronic medical history system and dynamically monitor the risk of individual patients, which enhanced the applicability. These findings underscore the model's potential for broader clinical application.

Several limitations must be acknowledged. First, the retrospective design and reliance on an external validation cohort limit the conclusions; a prospective, multicenter study with a larger dataset is needed. Second, the higher prevalence of MCI in individuals with T2DM in our study compared to other cohorts is likely due to the recruitment of older and more severely ill participants from hospital wards. As a result, potential confounders like age, glycemic variability, and hypoglycemic episodes must be carefully accounted for, as they could distort the interpretation of risk factors. Thus, the derived model is mainly applicable to the elder population with poorer glycemic control. Third, the external validation cohort was drawn from NHANES III, whose participants were significantly younger than the DECODE project, where only a small subset of patients underwent both neuropsychological testing and laboratory assessments. Besides, the NHANES cohort used an intuitively different cognitive assessment tool. Thus, this inconsistency of the diagnostic criteria would lead to biased evaluation of our diagnostic model. Although we used MoCA to evaluate general cognitive function, the NHANES III cohort employed different assessment criteria. This discrepancy in diagnostic standards may reduce the accuracy of our model's performance in external validation.

## 5. Conclusion

In summary, we developed a ML model to predict cognitive dysfunction in T2DM patients using accessible variables. This tool accurately identifies high-risk individuals for MCI and provides physicians with a user-friendly risk calculator for tailored prevention and treatment strategies. Future work will explore whether targeting these factors can prevent MCI onset, supporting early-stage intervention.

## Figures and Tables

**Table 1:** Demographical and clinical characteristics of eligible patients (grouped by the presence of MCI).

**Variables**	**T2DM-NC (** **n** = 1290**)**	**T2DM-MCI (** **n** = 784**)**	**p** ** value**
Demographics			
Age, years	57.9 ± 8.9	61.9 ± 8.4	< 0.001
Sex, female, *n* (%)	465 (31.3)	259 (40.7)	< 0.001
Education, years	12.6 ± 2.9	10.7 ± 3.2	< 0.001
Alcohol habits, *n* (%)	334 (25.9)	185 (23.6)	0.242
Smoking habits, *n* (%)	486 (37.7)	240 (30.3)	0.001
Diabetes-related indexes			
Diabetes duration (years)	9.6 ± 7.8	10.4 ± 8.2	0.043
HbA1c, %	8.8 ± 2.1	8.8 ± 2.2	0.141
FPG, mmol/L	8.3 ± 2.7	8.5 ± 2.7	0.026
2h-PG, mmol/L	15.6 ± 4.1	15.8 ± 4.4	0.359
FINS, uU/mL	9.0 ± 19.2	9.5 ± 24.6	0.644
2h-INS, uU/mL	39.6 ± 56.0	36.1 ± 50.3	0.154
FCP, pmol/L	627.8 ± 290.1	601.6 ± 302.4	0.055
2h-CP, pmol/L	1804.0 ± 1026.1	1654.4 ± 971.5	0.001
HOMA2-*β*	56.3 ± 33.5	52.6 ± 32.3	0.016
HOMA2-S	86.7 ± 96.3	89.1 ± 84.7	0.553
HOMA2-IR	1.6 ± 0.8	1.6 ± 0.8	0.115
Clinical and metabolic indexes			
BMI, kg/m^2^	24.8 ± 3.0	24.7 ± 3.2	0.242
WC, cm	91.0 ± 9.0	90.7 ± 9.3	0.554
HC, cm	97.8 ± 6.7	97.6 ± 8.5	0.607
WHR	0.9 ± 0.1	0.9 ± 0.1	0.980
SBP, mmHg	133.8 ± 17.1	135.2 ± 18.2	0.008
DBP, mmHg	82.4 ± 11.0	81.0 ± 11.2	0.088
ALT, mmol/L	25.7 ± 19.3	23.9 ± 17.3	0.039
AST, mmol/L	21.9 ± 12.6	21.2 ± 10.3	0.200
TG, mmol/L	1.7 ± 1.3	1.6 ± 1.0	0.018
TC, mmol/L	4.6 ± 1.2	4.6 ± 1.2	0.572
HDL-C, mmol/L	1.2 ± 0.3	1.3 ± 0.4	< 0.001
LDL-C, mmol/L	2.7 ± 1.0	2.7 ± 1.0	0.822
ApoA, mmol/L	1.1 ± 0.2	1.2 ± 0.2	< 0.001
ApoB, mmol/L	0.9 ± 0.3	0.8 ± 0.3	0.331
CREA, mmol/L	62.5 ± 16.8	61.8 ± 17.8	0.574
eGFR	119.7 ± 31.8	116.6 ± 32.0	0.039
UA, mmol/L	330.0 ± 85.2	320.8 ± 87.6	0.019
TSH, mmol/L	2.0 ± 1.3	2.1 ± 1.3	0.864
UACR	59.2 ± 274.9	68.8 ± 244.7	0.674
Vitamin B12, mmol/L	609.1 ± 323.5	619.6 ± 315.9	0.493
Complications and comorbidities, *n* (%)			
Diabetic peripheral neuropathy	432 (33.5)	344 (43.9)	< 0.001
Diabetes nephropathy	143 (11.1)	109 (13.9)	0.057
Diabetes retinopathy	245 (19.0)	141 (18.0)	0.568
Peripheral vascular disease	675 (52.4)	489 (56.1)	< 0.001
Cardiovascular disease	198 (15.3)	151 (19.3)	0.021
Cerebrovascular disease	131 (10.2)	133 (17.0)	< 0.001
Hypertension	619 (48.0)	447 (57.0)	< 0.001
Hyperlipidemia	597 (46.3)	375 (47.8)	0.492
MASLD	658 (51.0)	392 (50.0)	0.656
Osteoporosis	75 (5.8)	86 (11.0)	< 0.001
Family history, *n* (%)			
Family history of diabetes	605 (46.8)	350 (44.6)	0.551
Family history of hypertension	344 (26.7)	202 (25.8)	0.652

*Note:* Data are presented as mean ± standard deviation for continuous variables and *n* (%) for categorical variables. Comparisons of continuous variables between the two groups was analyzed by independent-samples *t*-test, while categorical variables were analyzed by chi-squared test. *p* value < 0.05 was considered significant.

Abbreviations: 2h-CP, 2-h postprandial C-peptide; 2h-INS, 2-h postprandial insulin; 2h-PG, 2-h postprandial plasma glucose; ALT, alanine aminotransferase; ApoA, apolipoprotein A; ApoB, apolipoprotein B; AST, aspartate aminotransferase; BMI, body mass index; CREA, creatinine; DBP, diastolic blood pressure; eGFR, estimated glomerular filtration rate; FCP, fasting C-peptide; FINS, fasting insulin; FPG, fasting plasma glucose; HbA1c, glycated hemoglobin; HC, hip circumference; HDL-C, high-density lipoprotein cholesterol; HOMA2-*β*, homeostatic model assessment of beta-cell function; HOMA2-IR, homeostatic model assessment of insulin resistance; HOMA2-S, homeostatic model assessment of insulin sensitivity; LDL-C, low-density lipoprotein cholesterol; MASLD, metabolic-associated steatotic liver disease; SBP, systolic blood pressure; TC, total cholesterol; TG, triglycerides; TSH, thyroid-stimulating hormone; UA, uric acid; UACR, urinary albumin-to-creatinine ratio; WC, waist circumference; WHR, waist-to-hip ratio.

**Table 2:** Predictive performance of machine learning algorithms on internal test dataset.

	**AUC**	**Accuracy**	**Recall**	**Precision**	**F**1** score**	**Brier score**
LR	0.73 ± 0.01	0.70 ± 0.03	0.47 ± 0.07	0.63 ± 0.05	0.54 ± 0.06	0.196
RF	0.73 ± 0.03	0.68 ± 0.01	0.40 ± 0.048	0.65 ± 0.02	0.54 ± 0.04	0.201
MLP	0.70 ± 0.03	0.66 ± 0.04	0.40 ± 0.15	0.62 ± 0.06	0.42 ± 0.08	0.206
AdaBoost	0.75 ± 0.02	0.70 ± 0.04	0.50 ± 0.06	0.63 ± 0.07	0.56 ± 0.06	0.221
CatBoost	0.74 ± 0.02	0.69 ± 0.01	0.64 ± 0.02	0.59 ± 0.02	0.61 ± 0.02	0.203
SVC	0.73 ± 0.03	0.66 ± 0.04	0.74 ± 0.09	0.54 ± 0.04	0.62 ± 0.06	0.201
AdaBoost (tuned)	0.76 ± 0.01	0.69 ± 0.02	0.43 ± 0.01	0.63 ± 0.04	0.51 ± 0.01	0.213
CatBoost (tuned)	0.73 ± 0.02	0.68 ± 0.02	0.59 ± 0.05	0.57 ± 0.03	0.58 ± 0.04	0.197
SVC (tuned)	0.74 ± 0.04	0.67 ± 0.03	0.75 ± 0.06	0.54 ± 0.03	0.63 ± 0.04	0.199

Abbreviations: AdaBoost, adaptive boosting; AUC, area under the curve; CatBoost, categorical boosting; LR, logistic regression; MLP, multilayer perceptron; RF, random forest; SVC, supportive vector classifier.

**Table 3:** Predictive performance of machine learning algorithms on external validation dataset from Validation Set 2.

	**AUC**	**Accuracy**	**Recall**	**Precision**	**F**1** score**	**Brier score**
AdaBoost (tuned)	0.78	0.55	0.68	0.76	0.66	0.23
CatBoost (tuned)	0.80	0.70	0.72	0.74	0.73	0.19
SVC (tuned)	0.80	0.75	0.89	0.72	0.80	0.22

Abbreviations: AdaBoost, adaptive boosting; AUC, area under the curve; CatBoost, categorical boosting; SVC, supportive vector classifier.

## Data Availability

The datasets generated and/or analyzed during the current study are not publicly available due to privacy issues of the medical records but are available from the corresponding authors on reasonable request.

## Supporting Information

Supporting Information Additional supporting information can be found online in the Supporting Information section. Figure S1: Variable importance and feature correlation for model feature selection. (A) Relative variable importance derived in random forest–based feature selection. (B) Correlation between features for feature elimination based on Pearson's correlation coefficients. Figure S2: Presentation of the web interface of our model. An example of a 67-year-old participant with 12 years of education, 5 years of history of diabetes, BMI of 25.71 kg/m^2^, SBP of 120 mmHg, FPG 8.0 mmol/L, PCP 2000 pmol/L, TG 3.21 mmol/L, TC 3.12 mmol/L, and ApoA 0.73 g/L; his risk of developing mild cognitive impairment was 58.0%. Table S1: Basic characteristics of participants. Table S2: Clinical characteristics of the validation dataset.
