# Supplementary material for: Predicting Mild Cognitive Impairment in Type 2 Diabetes: A Machine Learning Approach
Source: J Diabetes Res. 2025 Oct 19;2025:7304414. doi: 10.1155/jdr/7304414 (PMC12554921; doi:10.1155/jdr/7304414)
Supplement: Supporting Information — Additional supporting information can be found online in the Supporting Information section. Figure S1: Variable importance and feature correlation for model feature selection. (A) Relative variable importance derived in random forest–based feature selection. (B) Correlation between features for feature elimination based on Pearson's correlation coefficients. Figure S2: Presentation of the web interface of our model. An example of a 67-year-old participant with 12 years of education, 5 years of history of diabetes, BMI of 25.71 kg/m2, SBP of 120 mmHg, FPG 8.0 mmol/L, PCP 2000 pmol/L, TG 3.21 mmol/L, TC 3.12 mmol/L, and ApoA 0.73 g/L; his risk of developing mild cognitive impairment was 58.0%. Table S1: Basic characteristics of participants. Table S2: Clinical characteristics of the validation dataset. [file 7304414.f1.docx]

**Supplemental Materials**

Clinical features were obtained from the electronic health records, including 48 variables: demographic characteristics (age, sex, education, alcohol habits, smoking habits), laboratory variables (glycated hemoglobin[HbA1c], fasting plasma glucose[FPG], 2-hour postprandial plasma glucose[2h-PG], fasting insulin[FINS], 2-hour postprandial insulin[2h-INS], fasting C-peptide[FCP], 2-hour postprandial C-peptide[2h-CP], homeostatic model assessment of beta-cell function[HOMA2-β], homeostatic model assessment of insulin sensitivity[HOMA2-S], homeostatic model assessment of insulin resistance[HOMA2-IR], alanine aminotransferase[ALT], aspartate aminotransferase[AST], triglycerides[TG], total cholesterol[TC], high-density lipoprotein cholesterol[HDL-C], low-density lipoprotein cholesterol[LDL-C], apolipoprotein A[ApoA], Apolipoprotein B[ApoB], creatinine[CREA], estimated glomerular filtration rate[eGFR], uric acid[UA], thyroid stimulating hormone[TSH], urinary albumin-to-creatinine ratio[UACR]), physical measures (body mass index, waist circumference, hip circumference, waist-to-hip ratio, systolic blood pressure, diastolic blood pressure), diabetic complications and comorbidities (diabetic peripheral neuropathy, diabetes nephropathy, diabetes retinopathy, peripheral vascular disease, cardiovascular disease, cerebrovascular disease, hypertension, hyperlipidemia, metabolic dysfunction-associated fatty liver disease, osteoporosis), and medical and family history (diabetes duration, family history of diabetes, family history of hypertension). Among these variables, homeostasis model assessment-2(HOMA2) computational model was calculated with fasting serum C-peptide and plasma glucose values. The eGFR was calculated using the abbreviated Modification of Diet Renal Disease (MDRD) equation based on plasma creatinine and other demographic data.

Figure S1: Variable Importance and Feature Correlation for Model Feature Selection: (A) Relative variable importance derived in random forest-based feature selection; (B) Correlation between features for feature elimination based on Pearson’s correlation coefficients.


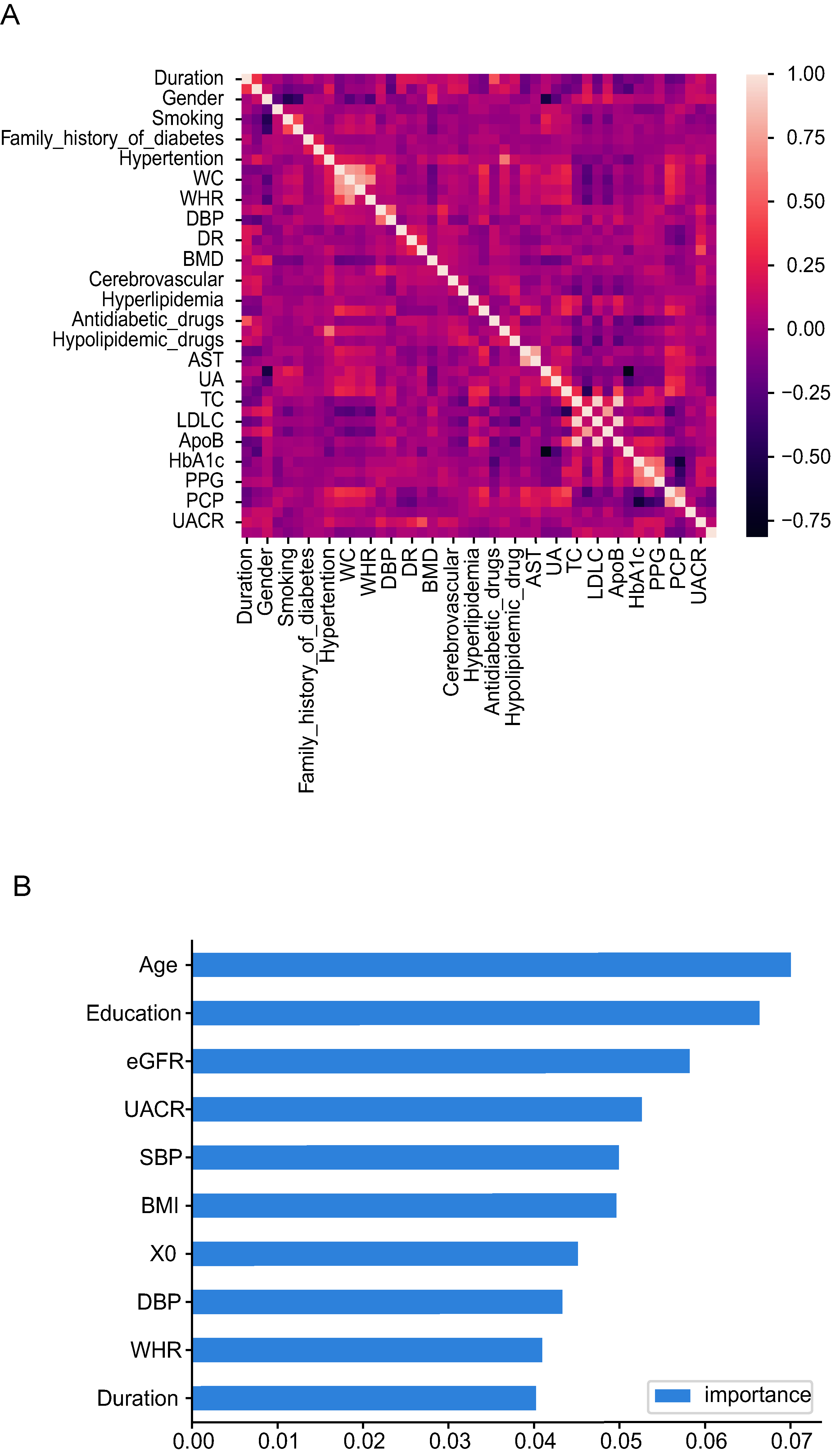


Figure S2:Presentation of the web interface of our model: An example of a 67-year-old participant with 12 years of education, five years of history of diabetes, BMI of 25.71 kg / m2, SBP of 120 mmHg, FPG 8.0 mmol / L, PCP 2000 pmol / L, TG 3.21 mmol / L, TC 3.12 mmol / L, and ApoA 0.73 g / L, his risk of developing mild cognitive impairment was 58.0%.


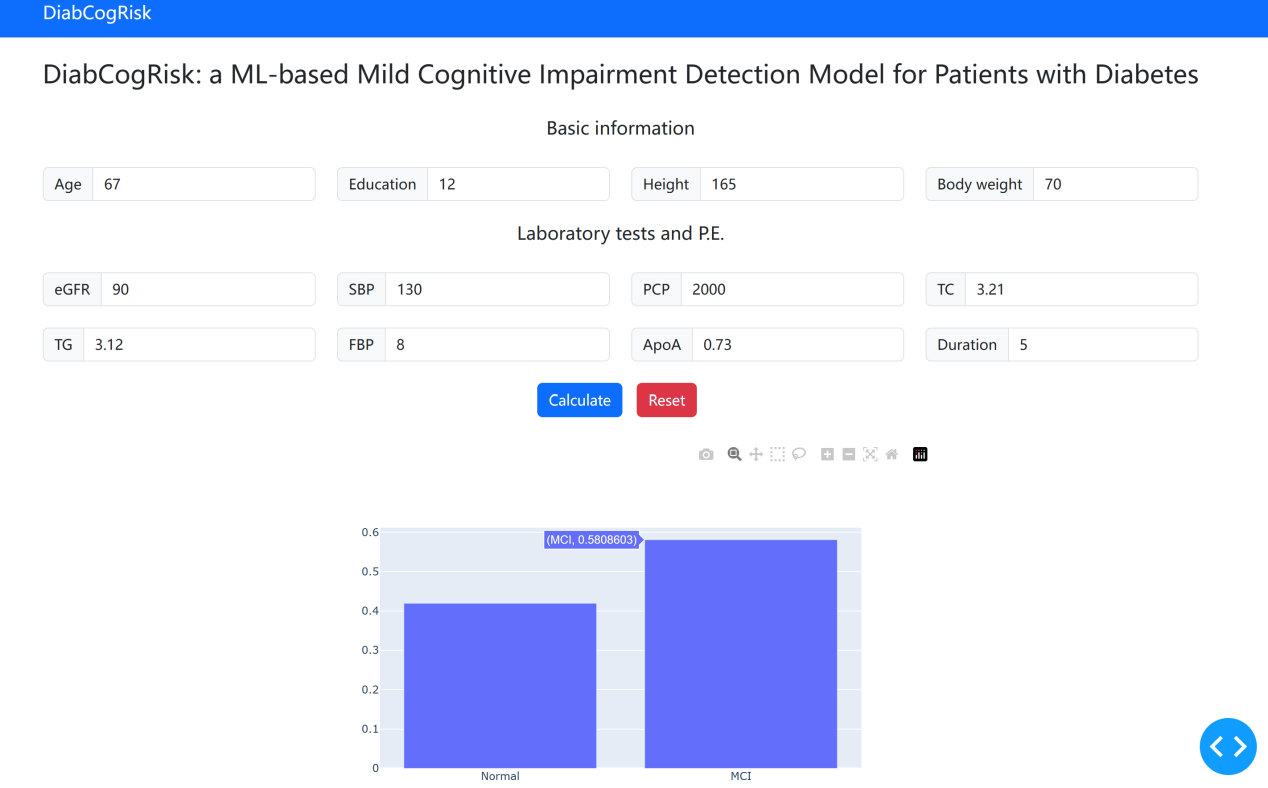


Table S1:Basic Characteristics of Participants

| Variables | Training set (n=1451) | Test set(n=623) | P value |
| --- | --- | --- | --- |
| Demographics |  |  |  |
| Age, years | 59.3±8.3 | 58.8±9.3 | 0.222 |
| Sex,female, n (%) | 511(35.2) | 213(34.2) | 0.653 |
| Education, years | 11.8±3.1 | 11.9±3.2 | 0.949 |
| Alcohol habits, n (%) | 369(25.4) | 150(24.1) | 0.514 |
| Smoking habits, n (%) | 503(34.7) | 223(35.8) | 0.621 |
| Diabetes-related indexes |  |  |  |
| Diabetes duration (years) | 10.1±7.9 | 9.5±8.0 | 0.092 |
| HbA1c, % | 8.7±2.0 | 8.9±2.1 | 0.057 |
| FPG, mmol/L | 8.3±2.7 | 8.5±2.7 | 0.128 |
| 2h-PG, mmol/L | 15.7±4.2 | 15.7±4.4 | 0.748 |
| FINS, uU/mL | 9.2±21.2 | 9.4±23.2 | 0.534 |
| 2h-INS, uU/mL | 37.6±54.9 | 34.1±56.1 | 0.220 |
| FCP, pmol/L | 613.2±294.1 | 627.5±296.1 | 0.316 |
| 2h-CP, pmol/L | 1746.±992.6 | 1749.6±1045.2 | 0.941 |
| HOMA2-β | 56.3±33.5 | 52.6±32.3 | 0.016 |
| HOMA2-S | 86.7±96.3 | 89.1±84.7 | 0.553 |
| HOMA2-IR | 1.6±0.8 | 1.6±0.8 | 0.115 |
| Clinical and metabolic indexes | | | |
| BMI, kg/m2 | 24.7±3. | 24.9±3.1 | 0.057 |
| WC, cm | 90.6±8.9 | 91.5±9.6 | 0.061 |
| HC, cm | 97.5±6.9 | 98.1±8.4 | 0.113 |
| WHR | 0.9±0.1 | 0.9±0.1 | 0.114 |
| SBP, mmHg | 134.5±17.5 | 134.1±17.7 | 0.609 |
| DBP, mmHg | 81.9±10.9 | 81.9±11.5 | 0.977 |
| ALT, mmol/L | 24.7±17.8 | 25.7±20.6 | 0.278 |
| AST, mmol/L | 21.7±12.4 | 21.6±11.7 | 0.305 |
| TG, mmol/L | 1.6±1.1 | 1.7±1.3 | 0.160 |
| TC, mmol/L | 4.6±1.2 | 4.6±1.2 | 0.372 |
| HDL-C, mmol/L | 1.2±0.3 | 1.2±0.3 | 0.154 |
| LDL-C, mmol/L | 2.7±1.0 | 2.7±1.0 | 0.643 |
| ApoA, mmol/L | 1.2±0.2 | 1.2±0.2 | 0.761 |
| ApoB, mmol/L | 0.8±0.3 | 0.9±0.3 | 0.606 |
| CREA, mmol/L | 62.9±20.3 | 63.1±18.8 | 0.847 |
| eGFR | 118.2±30.8 | 119.1±34.4 | 0.565 |
| UA, mmol/L | 326.2±85.3 | 327.3±88.5 | 0.785 |
| TSH, mmol/L | 2.1±1.4 | 2.±1.3 | 0.631 |
| UACR | 59.5±258.4 | 71.2±275.0 | 0.373 |
| Vitamin B12, mmol/L | 617.6±328.7 | 602.6±299.9 | 0.360 |
| Complications and comorbidities, n (%) | | | |
| Diabetic peripheral neuropathy | 553(38.1) | 223(35.8) | 0.317 |
| Diabetes nephropathy | 166(11.4) | 86(13.8) | 0.131 |
| Diabetes retinopathy | 269(18.5) | 117(18.8) | 0.897 |
| Peripheral vascular disease | 804(55.4) | 360 (57.8) | 0.263 |
| Cardiovascular disease | 251 (17.3) | 98 (15.7) | 0.382 |
| Cerebrovascular disease | 183(12.6) | 81 (13.0) | 0.807 |
| Hypertension | 748(51.6) | 318(51.0) | 0.832 |
| Hyperlipidemia | 671(46.2) | 301(48.3) | 0.386 |
| MASLD | 733(50.5) | 317(50.9) | 0.879 |
| Osteoporosis | 116(8.0) | 45(7.2) | 0.833 |
| Family history, n (%) |  |  |  |
| Family history of diabetes | 675(46.5) | 280(44.9) | 0.509 |
| Family history of hypertension | 378(26.1) | 168(27.0) | 0.664 |

Table S2: Clinical characteristics of the validation dataset

| Variables | Training set (n=2074) | Validation cohort 1  (n=196) | Validation cohort 2  (n=280) | p-value |
| --- | --- | --- | --- | --- |
| Age, years | 59.2±8.7 | 56.7±6.9^a^ | 49.4±5.9^ab^ | ＜0.001 |
| Male sex, n (%) | 724(34.9) | 82(50.9)^a^ | 153(54.6)^a^ | ＜0.001 |
| Education, years | 11.9±3.1 | 9.7±3.0^a^ | 9.9±4.1^a^ | ＜0.001 |
| Duration, years | 9.9±7.9 | 8.1±6.9^a^ | 7.3±8.5^a^ | ＜0.001 |
| BMI, kg/m2 | 24.8±3.0 | 26.2±3.8^a^ | 31.9±6.4^ab^ | ＜0.001 |
| SBP, mmHg | 134.4±17.5 | 132.5±17.3 | 133.0±20.5 | 0.269 |
| 2h-CP, pmol/L | 1747.1±1008.3 | 1482.7±966.8^a^ | 951.3±965.8^ab^ | ＜0.001 |
| FPG, mmol/L | 8.4±2.7 | 9.6±3.9^a^ | 9.5±4.5^a^ | ＜0.001 |
| TG, mmol/L | 1.6±1.2 | 2.1±1.8^a^ | 2.6±2.5^ab^ | ＜0.001 |
| TC, mmol/L | 4.6±1.2 | 4.6±1.2 | 5.6±1.2^ab^ | ＜0.001 |
| ApoA, mmol/L | 1.2±0.2 | 1.2±0.2 | 1.4±0.2^ab^ | ＜0.001 |
| eGFR | 118.5±31.9 | 112.0±28.5^a^ | 73.5±18.8^ab^ | ＜0.001 |

Note: Data are presented as mean ± standard deviation for continuous variables and n (%) for categorical variables. Comparisons between the three groups were performed using one-way analysis of variance (ANOVA) for continuous variables and chi-square (χ²) tests for categorical variables. For variables with significant differences across the groups, post hoc pairwise comparisons were conducted.

P value < 0.05 was considered significant.

^a^ Significance compared with Training set.

^b^ Significance compared with Validation cohort 1.

Abbreviations: BMI, body mass index; SBP, systolic blood pressure; FPG, fasting plasma glucose; 2h-CP, 2-hour postprandial C-peptide; TG, triglycerides; TC, total cholesterol; ApoA, apolipoprotein A; eGFR, estimated glomerular filtration rate.
